# Bromidotricarbon­yl[4-iodo-*N*-(pyridin-2-yl­methyl­idene)aniline-κ^2^ *N*,*N*′]rhenium(I)

**DOI:** 10.1107/S1600536810037104

**Published:** 2010-09-30

**Authors:** Saeed Dehghanpour, Ali Mahmoudi

**Affiliations:** a Department of Chemistry, Islamic Azad University, Karaj Branch, Karaj, Iran

## Abstract

In the title compound, [ReBr(C_12_H_9_IN_2_)(CO)_3_], the coordination geometry of the Re^I^ ion is a distorted *fac*-ReC_3_BrN_2_ octa­hedron, arising from the *N*,*N*′-bidentate ligand, a bromide ion and a facial arrangement of three carbonyl ligands. The dihedral angle between the aromatic rings in the 4-iodo-*N*-(pyridin-2-yl­methyl­idene)aniline ligand is 46.2 (3)°. The bromide ion and its corresponding *trans* CO mol­ecule are disordered over two sets of sites in a 0.966 (3):0.034 (3) ratio.

## Related literature

For the synthesis of the ligand, see: Dehghanpour *et al.* (2009*a* ▶). For background to diimine complexes and related structures see: Dehghanpour *et al.* (2009*b* ▶, 2010 ▶).
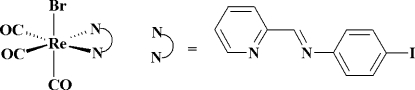


## Experimental

### 

#### Crystal data

[ReBr(C_12_H_9_IN_2_)(CO)_3_]
*M* *_r_* = 658.25
Triclinic, 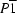

*a* = 8.8850 (4) Å
*b* = 9.0239 (4) Å
*c* = 10.9736 (4) Å
α = 75.202 (2)°
β = 80.885 (3)°
γ = 84.668 (3)°
*V* = 838.64 (6) Å^3^
*Z* = 2
Mo *K*α radiation
μ = 11.48 mm^−1^
*T* = 150 K
0.08 × 0.07 × 0.03 mm

#### Data collection

Nonius KappaCCD diffractometer
Absorption correction: multi-scan (*SORTAV*; Blessing, 1995 ▶) *T*_min_ = 0.463, *T*_max_ = 0.707
9719 measured reflections
3796 independent reflections
3256 reflections with *I* > 2σ(*I*)
*R*_int_ = 0.059

#### Refinement

*R*[*F* ^2^ > 2σ(*F* ^2^)] = 0.041
*wR*(*F* ^2^) = 0.108
*S* = 1.04
3796 reflections
212 parameters
1 restraint
H-atom parameters constrained
Δρ_max_ = 2.74 e Å^−3^
Δρ_min_ = −2.80 e Å^−3^

### 

Data collection: *COLLECT* (Nonius, 2002 ▶); cell refinement: *DENZO-SMN* (Otwinowski & Minor, 1997 ▶); data reduction: *DENZO-SMN*; program(s) used to solve structure: *SIR92* (Altomare *et al.*, 1994 ▶); program(s) used to refine structure: *SHELXTL* (Sheldrick, 2008 ▶); molecular graphics: *PLATON* (Spek, 2009 ▶); software used to prepare material for publication: *SHELXTL*.

## Supplementary Material

Crystal structure: contains datablocks I, global. DOI: 10.1107/S1600536810037104/hb5621sup1.cif

Structure factors: contains datablocks I. DOI: 10.1107/S1600536810037104/hb5621Isup2.hkl

Additional supplementary materials:  crystallographic information; 3D view; checkCIF report

## Figures and Tables

**Table 1:** Selected bond lengths (Å)

Re1—C2	1.919 (7)
Re1—C3	1.924 (8)
Re1—C1	1.928 (11)
Re1—N1	2.179 (6)
Re1—N2	2.188 (5)
Re1—Br1	2.6139 (8)

**Fig. 1.**
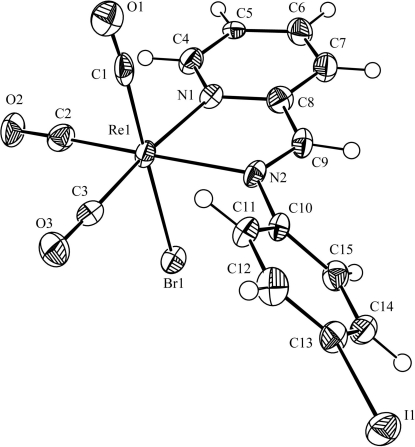
A view of the structure of the title complex, with displacement ellipsoids drawn at the 50% probability level [H atoms are represented as spheres of arbitrary radius].

## supplementary crystallographic information

### Comment

In our ongoing studies on the synthesis, structural and spectroscopic characterization of transition metal complexes with diimine ligands (Dehghanpour *et al.*, 2009*a*; Dehghanpour *et al.*, 2010), here we report crystal structure of the title complex. The title complex, (I), Fig. 1, was prepared by the reaction of Re(CO)_5_Br with the bidentate ligand (4-iodophenyl)pyridin-2-ylmethyleneamine (Dehghanpour *et al.*, 2009*b*);(Scheme I).

The rhenium atom is coordinated by the N1 pyridine and N2 imine atoms, affording a five-membered chelate ring, as well as three carbonyl carbon atoms and a bromide atom. The resulting coordination geometry can be described as distorted octahedral [the main distortion being the N1–Re1–N2 and C3–Re1–N2 angles]. The rhenium–carbonyl bond lengths do not show any significant differences and The Re–N bond lengths are similar and within the range expected for such complexes.

### Experimental

A mixture of [Re(CO)_5_Br] (406 mg, 1 mmol) and ligand (308 mg, 1 mmol) in dry, degassed toluene (30 *cm*3) was heated to reflux for 4 h under N_2_ to give a bright red solution. The solvent was removed under vacuum and the crude material recrystallized from CH_2_Cl_2_/hexane to give red blocks of (I). Yield: 89%. Calc. for C_15_H_9_BrIN_2_O_3_Re: C 27.37, H 1.37, N 4.26%; found: C 27.30, H 1.42, N 4.20%.

### Refinement

The H(C) atom positions were calculated and refined in isotropic approximatiom within riding model with the *U*_iso_(H) parameters equal to 1.2 *U*_eq_(Ci) where U(Ci) is the equivalent thermal parameters of the carbon atoms to which corresponding H atoms are bonded The Br atom is disordered over two sites in a 0.966 (3):0.034 (3) ratio; the corresponding carbonyl ligand must also be disordered in the reverse sense, but the occurancies of the minor disorder component were too small to model, so C1 and O1 were refined with full occupancies.

### Crystal data

**Table:**

[ReBr(C_12_H_9_IN_2_)(CO)_3_]	*Z* = 2
*M**_r_* = 658.25	*F*(000) = 600
Triclinic, *P*1	*D*_x_ = 2.607 Mg m^−^^3^
Hall symbol: -P 1	Mo *K*α radiation, λ = 0.71073 Å
*a* = 8.8850 (4) Å	Cell parameters from 9719 reflections
*b* = 9.0239 (4) Å	θ = 2.7–27.5°
*c* = 10.9736 (4) Å	µ = 11.48 mm^−^^1^
α = 75.202 (2)°	*T* = 150 K
β = 80.885 (3)°	Block, red
γ = 84.668 (3)°	0.08 × 0.07 × 0.03 mm
*V* = 838.64 (6) Å^3^	

### Data collection

**Table:**

Nonius KappaCCD diffractometer	3796 independent reflections
Radiation source: fine-focus sealed tube	3256 reflections with *I* > 2σ(*I*)
graphite	*R*_int_ = 0.059
Detector resolution: 9 pixels mm^-1^	θ_max_ = 27.5°, θ_min_ = 2.7°
φ scans and ω scans with κ offsets	*h* = −10→11
Absorption correction: multi-scan (*SORTAV*; Blessing, 1995)	*k* = −11→11
*T*_min_ = 0.463, *T*_max_ = 0.707	*l* = −12→14
9719 measured reflections	

### Refinement

**Table:**

Refinement on *F*^2^	Primary atom site location: structure-invariant direct methods
Least-squares matrix: full	Secondary atom site location: difference Fourier map
*R*[*F*^2^ > 2σ(*F*^2^)] = 0.041	Hydrogen site location: inferred from neighbouring sites
*wR*(*F*^2^) = 0.108	H-atom parameters constrained
*S* = 1.04	*w* = 1/[σ^2^(*F*_o_^2^) + (0.0591*P*)^2^ + 1.7708*P*] where *P* = (*F*_o_^2^ + 2*F*_c_^2^)/3
3796 reflections	(Δ/σ)_max_ = 0.001
212 parameters	Δρ_max_ = 2.74 e Å^−^^3^
1 restraint	Δρ_min_ = −2.80 e Å^−^^3^

### Special details

**Table:**

Experimental. multi-scan from symmetry-related measurements SORTAV (Blessing 1995)
Geometry. All e.s.d.'s (except the e.s.d. in the dihedral angle between two l.s. planes) are estimated using the full covariance matrix. The cell e.s.d.'s are taken into account individually in the estimation of e.s.d.'s in distances, angles and torsion angles; correlations between e.s.d.'s in cell parameters are only used when they are defined by crystal symmetry. An approximate (isotropic) treatment of cell e.s.d.'s is used for estimating e.s.d.'s involving l.s. planes.
Refinement. Refinement of *F*^2^ against ALL reflections. The weighted *R*-factor *wR* and goodness of fit *S* are based on *F*^2^, conventional *R*-factors *R* are based on *F*, with *F* set to zero for negative *F*^2^. The threshold expression of *F*^2^ > σ(*F*^2^) is used only for calculating *R*-factors(gt) *etc*. and is not relevant to the choice of reflections for refinement. *R*-factors based on *F*^2^ are statistically about twice as large as those based on *F*, and *R*- factors based on ALL data will be even larger.

### Fractional atomic coordinates and isotropic or equivalent isotropic displacement parameters (Å^2^)

**Table:**

	*x*	*y*	*z*	*U*_iso_*/*U*_eq_	Occ. (<1)
Re1	0.71427 (3)	0.42431 (3)	0.25069 (2)	0.02270 (11)	
I1	1.35429 (6)	−0.18136 (6)	0.09158 (5)	0.03272 (15)	
Br1	0.77459 (9)	0.28546 (8)	0.47903 (6)	0.0279 (2)	0.966 (3)
O1	0.6470 (7)	0.5896 (8)	−0.0170 (7)	0.0374 (14)	
O2	0.6352 (7)	0.7283 (6)	0.3297 (6)	0.0395 (13)	
O3	1.0434 (6)	0.5242 (6)	0.1639 (5)	0.0387 (13)	
N1	0.4879 (7)	0.3344 (6)	0.3111 (5)	0.0222 (12)	
N2	0.7420 (7)	0.1892 (6)	0.2274 (5)	0.0224 (12)	
C1	0.6686 (12)	0.5243 (11)	0.0821 (10)	0.028 (2)	
C2	0.6644 (8)	0.6156 (8)	0.2979 (7)	0.0293 (16)	
C3	0.9211 (9)	0.4851 (8)	0.1974 (7)	0.0270 (15)	
C4	0.3602 (9)	0.4075 (8)	0.3539 (7)	0.0293 (16)	
H4A	0.3651	0.5104	0.3590	0.035*	
C5	0.2205 (7)	0.3394 (7)	0.3913 (6)	0.0191 (13)	
H5A	0.1333	0.3936	0.4244	0.023*	
C6	0.2107 (9)	0.1929 (9)	0.3795 (7)	0.0318 (17)	
H6A	0.1153	0.1463	0.4006	0.038*	
C7	0.3416 (9)	0.1134 (9)	0.3365 (7)	0.0323 (17)	
H7A	0.3381	0.0109	0.3299	0.039*	
C8	0.4770 (9)	0.1874 (8)	0.3036 (6)	0.0279 (15)	
C9	0.6197 (8)	0.1133 (8)	0.2602 (6)	0.0258 (15)	
H9A	0.6234	0.0092	0.2559	0.031*	
C10	0.8834 (8)	0.1079 (7)	0.1978 (6)	0.0217 (14)	
C11	0.9899 (8)	0.1744 (8)	0.0970 (6)	0.0257 (15)	
H11A	0.9705	0.2764	0.0495	0.031*	
C12	1.1240 (9)	0.0943 (9)	0.0646 (7)	0.0310 (16)	
H12A	1.1953	0.1396	−0.0062	0.037*	
C13	1.1535 (8)	−0.0534 (8)	0.1370 (7)	0.0261 (15)	
C14	1.0489 (9)	−0.1201 (8)	0.2407 (6)	0.0274 (15)	
H14A	1.0700	−0.2207	0.2901	0.033*	
C15	0.9143 (9)	−0.0397 (8)	0.2718 (6)	0.0269 (15)	
H15A	0.8431	−0.0844	0.3429	0.032*	
Br1A	0.652 (6)	0.551 (5)	0.040 (3)	0.030*	0.034 (3)

### Atomic displacement parameters (Å^2^)

**Table:**

	*U*^11^	*U*^22^	*U*^33^	*U*^12^	*U*^13^	*U*^23^
Re1	0.02352 (18)	0.01442 (16)	0.02889 (18)	−0.00057 (11)	−0.00101 (12)	−0.00477 (11)
I1	0.0290 (3)	0.0314 (3)	0.0382 (3)	0.0068 (2)	−0.0042 (2)	−0.0126 (2)
Br1	0.0331 (4)	0.0230 (4)	0.0266 (4)	0.0010 (3)	−0.0037 (3)	−0.0056 (3)
O1	0.038 (3)	0.034 (4)	0.038 (4)	−0.002 (3)	−0.002 (3)	−0.007 (3)
O2	0.046 (3)	0.021 (3)	0.055 (3)	0.001 (2)	−0.008 (3)	−0.015 (2)
O3	0.027 (3)	0.035 (3)	0.051 (3)	−0.011 (2)	−0.001 (3)	−0.004 (3)
N1	0.024 (3)	0.019 (3)	0.022 (3)	0.001 (2)	−0.002 (2)	−0.003 (2)
N2	0.031 (3)	0.015 (3)	0.020 (3)	0.004 (2)	−0.003 (2)	−0.006 (2)
C1	0.023 (4)	0.016 (5)	0.047 (6)	−0.002 (3)	0.000 (5)	−0.016 (4)
C2	0.024 (4)	0.026 (4)	0.035 (4)	0.002 (3)	−0.001 (3)	−0.004 (3)
C3	0.028 (4)	0.021 (3)	0.030 (4)	0.002 (3)	−0.009 (3)	−0.002 (3)
C4	0.031 (4)	0.022 (4)	0.034 (4)	0.003 (3)	−0.005 (3)	−0.006 (3)
C5	0.015 (3)	0.022 (3)	0.019 (3)	0.001 (3)	−0.005 (2)	−0.003 (2)
C6	0.031 (4)	0.030 (4)	0.033 (4)	−0.011 (3)	−0.004 (3)	−0.002 (3)
C7	0.040 (4)	0.024 (4)	0.034 (4)	−0.005 (3)	−0.007 (3)	−0.006 (3)
C8	0.031 (4)	0.028 (4)	0.026 (3)	0.001 (3)	−0.004 (3)	−0.009 (3)
C9	0.033 (4)	0.017 (3)	0.027 (3)	−0.006 (3)	−0.002 (3)	−0.004 (3)
C10	0.026 (3)	0.015 (3)	0.024 (3)	0.000 (3)	−0.002 (3)	−0.007 (3)
C11	0.029 (4)	0.018 (3)	0.029 (3)	0.003 (3)	−0.003 (3)	−0.005 (3)
C12	0.032 (4)	0.032 (4)	0.028 (4)	−0.007 (3)	0.007 (3)	−0.008 (3)
C13	0.025 (4)	0.025 (4)	0.031 (4)	0.004 (3)	0.000 (3)	−0.016 (3)
C14	0.035 (4)	0.022 (3)	0.025 (3)	0.003 (3)	−0.008 (3)	−0.006 (3)
C15	0.035 (4)	0.023 (3)	0.022 (3)	−0.005 (3)	−0.004 (3)	−0.003 (3)

### Geometric parameters (Å, °)

**Table:**

Re1—C2	1.919 (7)	C5—C6	1.373 (10)
Re1—C3	1.924 (8)	C5—H5A	0.9500
Re1—C1	1.928 (11)	C6—C7	1.391 (11)
Re1—N1	2.179 (6)	C6—H6A	0.9500
Re1—N2	2.188 (5)	C7—C8	1.383 (11)
Re1—Br1A	2.43 (3)	C7—H7A	0.9500
Re1—Br1	2.6139 (8)	C8—C9	1.447 (10)
I1—C13	2.096 (7)	C9—H9A	0.9500
O1—Br1A	0.64 (3)	C10—C11	1.383 (9)
O1—C1	1.133 (13)	C10—C15	1.400 (9)
O2—C2	1.153 (9)	C11—C12	1.380 (10)
O3—C3	1.148 (9)	C11—H11A	0.9500
N1—C4	1.339 (9)	C12—C13	1.392 (10)
N1—C8	1.364 (9)	C12—H12A	0.9500
N2—C9	1.291 (9)	C13—C14	1.394 (10)
N2—C10	1.430 (9)	C14—C15	1.381 (10)
C1—Br1A	0.50 (3)	C14—H14A	0.9500
C4—C5	1.391 (10)	C15—H15A	0.9500
C4—H4A	0.9500		
			
C2—Re1—C3	88.3 (3)	C6—C5—C4	119.0 (6)
C2—Re1—C1	88.8 (4)	C6—C5—H5A	120.5
C3—Re1—C1	88.9 (4)	C4—C5—H5A	120.5
C2—Re1—N1	96.6 (3)	C5—C6—C7	119.3 (7)
C3—Re1—N1	174.8 (3)	C5—C6—H6A	120.3
C1—Re1—N1	92.7 (3)	C7—C6—H6A	120.3
C2—Re1—N2	169.6 (3)	C8—C7—C6	118.3 (7)
C3—Re1—N2	99.9 (3)	C8—C7—H7A	120.8
C1—Re1—N2	97.5 (3)	C6—C7—H7A	120.8
N1—Re1—N2	75.0 (2)	N1—C8—C7	123.0 (7)
C2—Re1—Br1A	88.4 (12)	N1—C8—C9	114.5 (6)
C3—Re1—Br1A	89.8 (12)	C7—C8—C9	122.4 (7)
C1—Re1—Br1A	1.0 (14)	N2—C9—C8	119.8 (6)
N1—Re1—Br1A	91.9 (12)	N2—C9—H9A	120.1
N2—Re1—Br1A	97.8 (12)	C8—C9—H9A	120.1
C2—Re1—Br1	91.8 (2)	C11—C10—C15	119.9 (6)
C3—Re1—Br1	91.6 (2)	C11—C10—N2	120.4 (6)
C1—Re1—Br1	179.2 (3)	C15—C10—N2	119.7 (6)
N1—Re1—Br1	86.79 (14)	C12—C11—C10	120.8 (6)
N2—Re1—Br1	81.81 (14)	C12—C11—H11A	119.6
Br1A—Re1—Br1	178.6 (12)	C10—C11—H11A	119.6
Br1A—O1—C1	6(5)	C11—C12—C13	119.2 (6)
C4—N1—C8	117.2 (6)	C11—C12—H12A	120.4
C4—N1—Re1	127.3 (5)	C13—C12—H12A	120.4
C8—N1—Re1	115.5 (5)	C12—C13—C14	120.5 (6)
C9—N2—C10	118.1 (6)	C12—C13—I1	121.0 (5)
C9—N2—Re1	115.1 (5)	C14—C13—I1	118.5 (5)
C10—N2—Re1	126.1 (4)	C15—C14—C13	119.9 (7)
Br1A—C1—O1	7(7)	C15—C14—H14A	120.0
Br1A—C1—Re1	175 (7)	C13—C14—H14A	120.0
O1—C1—Re1	176.0 (8)	C14—C15—C10	119.6 (6)
O2—C2—Re1	178.0 (7)	C14—C15—H15A	120.2
O3—C3—Re1	178.5 (7)	C10—C15—H15A	120.2
N1—C4—C5	123.0 (7)	C1—Br1A—O1	167 (10)
N1—C4—H4A	118.5	C1—Br1A—Re1	4(6)
C5—C4—H4A	118.5	O1—Br1A—Re1	170 (6)
